# A Longitudinal Study of the Human Oropharynx Microbiota Over Time Reveals a Common Core and Significant Variations With Self-Reported Disease

**DOI:** 10.3389/fmicb.2020.573969

**Published:** 2021-01-21

**Authors:** Lydia Luise Bach, Asha Ram, Umer Z. Ijaz, Thomas J. Evans, Jan Lindström

**Affiliations:** ^1^Institute of Biodiversity, Animal Health and Comparative Medicine, University of Glasgow, Glasgow, United Kingdom; ^2^School of Engineering, University of Glasgow, Glasgow, United Kingdom; ^3^Institute of Infection, Immunity and Inflammation, Glasgow Biomedical Research Centre, University of Glasgow, Glasgow, United Kingdom

**Keywords:** microbiome, core, oropharynx, oral, microbial, community

## Abstract

Our understanding of human microbial communities, in particular in regard to diseases is advancing, yet the basic understanding of the microbiome in healthy subjects over time remains limited. The oropharynx is a key target for colonization by several important human pathogens. To understand how the oropharyngeal microbiome might limit infections, and how intercurrent infections might be associated with its composition, we characterized the oropharyngeal microbiome of 18 healthy adults, sampled weekly over a 40-weeks using culture-independent molecular techniques. We detected nine phyla, 202 genera and 1438 assignments on OTU level, dominated by Firmicutes, Bacteroidetes, and Proteobacteria on phylum level. Individual microbiomes of participants were characterized by levels of high alpha diversity (mean = 204.55 OTUs, sd = 35.64), evenness (19.83, sd = 9.74) and high temporal stability (mean Pearson’s correlation between samples of 0.52, sd = 0.060), with greater differences in microbiome community composition between than within individuals. Significant changes in community composition were associated with disease states, suggesting that it is possible to detect specific changes in OTU abundance and community composition during illness. We defined the common core microbiota by varying occurrence and abundance thresholds showing that individual core microbiomes share a substantial number of OTUs across participants, chiefly *Streptococci* and *Veillonella*. Our results provide insights into the microbial communities that characterize the healthy human oropharynx, community structure and variability, and provide new approaches to define individual and shared cores. The wider implications of this result include the potential for modeling the general dynamics of oropharynx microbiota both in health and in response to antimicrobial treatments or probiotics.

## Introduction

Only about half of the approximately 60 trillion cells found within our bodies are of human origin, the rest comprises bacterial cells known as the microbiota (Sender et al., 2016). These microbial communities inhabit a wide number of habitats on the human body, such as the oral cavity, skin, vagina, or mucosal gut surface, providing important functions such as resisting pathogen invasion, regulating metabolism, and supporting the host’s immune system (Dethlefsen et al., 2007; Parfrey et al., 2011).

The oral cavity provides a variety of habitats to microbial communities, including teeth, gingival sulcus, tongue, cheek, lip, and contiguous with the oral cavity, tonsils, pharynx, and esophagus. Much of the prior work on these microbial communities has focused on the oral cavity microbiota as a single habitat (Aas et al., 2005; Jenkinson and Lamont, 2005; Gioula et al., 2018), showing that it is species-rich, with 500–700 species (Wilson et al., 1997; Paster et al., 2001), making it the most taxon-rich body site (Jenkinson, 2011; Huse et al., 2012; Faner et al., 2017).

The oral cavity is an entry point to the human body, and microbial taxa colonizing the oral cavities can spread via epithelial surfaces to other body sites such as the stomach, intestinal tract, trachea, or lungs. In addition microbes are involved in diseases such as caries (oral decay) (Gross et al., 2010; Tanner et al., 2018), periodontitis (gum disease) (Joshipura et al., 2003; Schulz et al., 2019), endodontic infections (root canal) (Brito et al., 2020), or tonsillitis (Lemon et al., 2010; Galli et al., 2020), and have been increasingly linked to systemic diseases such as cardiovascular disease (Beck and Offenbacher, 2005; Blekkenhorst et al., 2018), stroke (Joshipura et al., 2003), diabetes (Genco et al., 2005; Long et al., 2017), or pneumonia (Seymour et al., 2007; Awano et al., 2008; Mammen et al., 2020). Recently it has become apparent that, rather than being caused by single organisms, many of these infections are linked to communities of organisms, often occurring in complex biofilms (Hall-Stoodley et al., 2004; Jenkinson and Lamont, 2005; Bai et al., 2019). This has resulted in a shift of focus away from single species to considering that increasing understanding of human health and disease requires the characterization of the microbial community.

Dewhirst et al. (2010); Jenkinson (2011), and Verma et al. (2018) Microbial communities and their ecology have been described in several oral habitats such as teeth (Becker et al., 2002; Rademacher et al., 2019), tongue (Jiang et al., 2012; Wilbert et al., 2020), gingival sulcus (Paster et al., 2001; Wei et al., 2020), and saliva (Sakamoto et al., 2000; Nasidze et al., 2009), but with a particular focus on disease-state (Aas et al., 2005; Zaura et al., 2009) and, unfortunately, often lacking sufficient depth and spatial or temporal sampling breadth to unravel the ecology and dynamics of microbial communities (Yang et al., 2016). Moreover, less information is available regarding the healthy microbiome of the human oropharynx.

Another aspect that has gathered momentum, is the identification of a core microbiome, which has been an important goal of the Human Microbiome Project (Shetty et al., 2017; Edouard et al., 2018; Li and Ma, 2020). The core microbiome is defined as a suite of shared members within microbial consortia occurring in similar or the same habitat (Shade and Handelsman, 2012), and, as some authors argue, shared among all or the majority of humans (Turnbaugh et al., 2007; Hu et al., 2013). The core microbiome is likely to provide important ecosystem functions, and may therefore play a significant role in maintaining health, and can provide a deeper understanding of the dynamics and stability of the microbial community (Shade and Handelsman, 2012; Li and Ma, 2020). While some have argued that a core oral microbiome exists based on commonality of microbial taxa between participants (Zaura et al., 2009), the small numbers of participants, and data only representing snapshots, rather than longitudinal data, hamper the ability to generalize these conclusions (Jenkinson, 2011; Shetty et al., 2017).

In this study, we set out to define the general microbial community and phylogeny of the most prevalent taxa in the human oropharynx, its temporal dynamics and variability in community structure and to describe the core microbiome shared across baseline-healthy participants.

## Materials and Methods

### Participants and Sample Collection

Eighteen participants were recruited for this study, representing both genders (11 females, 7 males) ranging in age from 18 to 40 (mean 24.40, sd = 6.04), and were healthy (without illnesses, undergoing treatments, and non-smokers) and not on any long-term medication. The study was approved by the University of Glasgow Ethics Committee and consent was obtained (Ethics Application 2012107 and 200140021).

Participants were provided with Sigma Transwabs swabs in liquid amies (Medical Wire Ltd., United Kingdom) for bacterial detection, with samples taken from the tonsils and the posterior wall to tonsil. Participants were asked to take a swab once weekly, early in the morning prior brushing their teeth and breakfast, and kept a diary providing information about their health status. Samples were collected weekly, and bacterial swabs were processed typically within 2 h after collection. Sampling occurred in Glasgow, United Kingdom, between January and May and September and December in 2013.

### DNA Extractions

DNA was extracted using the QIAamp DNA Mini kit (Qiagen Ltd., United Kingdom) following the bacteria, swab, and tissue protocol (Salter et al., 2014). Extracted DNA was quantified using the Qubit 2.0 (Thermo Fisher Scientific, Q32866) and picogreen HS DNA assay (Invitrogen Ltd., United Kingdom). A volume of DNA (5 μl) was mixed with 2 μl of loading dye on a 1% agarose gel (1 g agarose to 100 ml TBE) along with a 1 Kb Invitrogen DNA ladder and ran at 100 v for 60 min to check purity. The DNA was then stored at −20°C until required. The details on processing the samples are given in Chapter 2 of http://theses.gla.ac.uk/8163/ and provided in Supplementary Material 1 (QIA-AMP DNA extraction protocol), Supplementary Material 2 (Production of an rDNA clone library), and Supplementary Material 3 (QIAGEN QIA gel extraction kit protocol).

### Bioinformatics

Trimming and filtering of paired-end sequencing reads was done using Sickle (version 1.2) by applying a sliding window approach and trimming regions where the average base quality drops below 20 (Joshi and Fass, 2011). This applied a 10 bp length threshold to discard reads that fall below this length. BayesHammer (Nikolenko et al., 2013) was used from the SPAdes assembler (version 2.5) to error correct the paired-end reads followed by PANDAseq (version 2.4) with a minimum overlap of 50 bp to assemble the forward and reverse reads into a single sequence spanning the entire V1–V2 region (Masella et al., 2012). The above choice of software showed a reduction in substitution errors by 77–98% with an average of 93.2% for MiSeq datasets (Schirmer et al., 2015). After having obtained the consensus sequences from each sample, UPARSE (version 7.0.1001) was used for OTU construction (Edgar, 2013). The approach pools together the reads from different samples and adds barcodes to keep an account of the samples these reads originate from. The reads are then dereplicated and sorted by decreasing abundance and discarding singletons. In the next step, the reads are clustered based on 97% similarity, discarding any reads that are shorter than 32 bp. The original barcoded reads were then matched against OTUs with 97% similarity to generate OTU tables for different samples. OTU representative sequences were then taxonomically classified against the RDP database using the standalone RDP classifier (version 2.6) (Wang et al., 2007). Phylogenetic distances between OTUs were produced by first using MAFFT (version 7.040) (Katoh and Standley, 2013) to align the OTUs against each other and then by using FastTree (version 2.1.7) on these alignments to generate an approximately maximum-likelihood phylogenetic tree (Price et al., 2010). Chimeras were removed from the most abundant reads, and a reference based using Gold database (Schirmer et al., 2015; D’Amore et al., 2016). Traditional pipelines were modified to get the optimum accuracy for amplicons based on benchmarking studies (Schirmer et al., 2015; D’Amore et al., 2016; Gerasimidis et al., 2016). The OTU table, phylogenetic tree, taxonomic information, and metadata were then used in multivariate statistical analysis. Even though the *de novo* chimera removal step removes reads that have chimeric models built from more abundant reads, a few chimeras may be missed, especially if they have parents that are absent from the reads or are present in very low abundance. Therefore, in the next step, we used a reference-based chimera filtering step using a gold database^1^ that is derived from the ChimeraSlayer reference database in the Broad Microbiome Utilities^2^.

### Statistical Analysis

All samples that contained less than 5000 reads were discarded in the analysis to allow comparison of all samples with sufficient statistical power. The relative abundance of taxa for each sample was calculated by dividing the read counts of that taxa by sample size and ranges from 0 to 1. Statistical analysis was performed in R software (version 3.1.2). Where appropriate before specific analyses, the abundance data was normalized (McMurdie and Holmes, 2013).

Hierarchical clustering was used to group samples according to their Pearson correlation distance, based on log10 + 1 transformed abundance data, providing a measure of inter and intra individual similarity of the microbial community.

Estimating alpha diversity and evenness is particularly challenging in microbial communities (Hughes et al., 2001). To do so, samples were bootstrapped to a minimum of 5000 reads with replacement to subsample at a standardized sampling effort. This resulted in estimates of average species richness and evenness from 100 trials per sample. Alpha diversity describes the count of unique OTUs in each sample, while evenness is inverse Simpson (Smith and Wilson, 1996), i.e., the proportional abundances of OTUs in each sample. Data was analyzed using a one-way ANOVA and Tukey *post hoc* test, after checking the assumptions.

### Multidimensional Scaling and Core Microbiome

To illustrate differences in bacterial community composition over time, two participants were selected; the first participant reporting no health issues, and the second reporting cold symptoms in two separate weeks within the study period. Participants were selected based on those with the largest number of samples available. Differences in community structure at phylum and OTU level (selecting the 20 most abundant OTUs) are shown, and the structure of the microbial community was assessed using a non-metric multidimensional scaling plot (NMDS) at OTU level for each participant with Bray-Curtis dissimilarity index (Kruskal, 1964). The overlaid vectors indicate significant correlations among the relative abundances of OTUs with the two axes of the NMDS using package vegan in r (Oksanen et al., 2013, 2018). All samples were bootstrapped samples to a minimum number of 5000 reads with replacement to subsample at a standardized sample effort and sampled 100 times. We used hierarchical cluster analysis in r to distinguish between three sample clusters, using hclust function in the stats package (R Core Team and C Worldwide, 2002), performing hierarchical clustering based on average distances from the distance matrix.

There has been no single standardized approach to identify the core microbiome, with most studies reporting the core based on presence/absence of taxa at specific threshold values of abundance and prevalence (Turnbaugh et al., 2007). Here, we analyzed the core microbiome using adjustable parameters for abundance (i.e., % of relative abundance in the sample, ranging from 5 to 100%) and prevalence (% of occurrence within samples), as developed previously providing a more integrated approach to view the core microbiome (Jalanka-Tuovinen et al., 2011). We subsequently used the heat_tree_matrix() function in the metacoder package (Foster et al., 2017) to establish significantly different relative abundances in core OTUs (based on log_2_ median ratio) between participants using a Wilcoxon test.

## Results

A total of 313 samples from 18 participants representing both genders (11 females, 7 males) ranging in age from 18 to 40 (mean 24.40, sd = 6.04) were analyzed. The taxonomic profiles across all participants revealed a total of nine phyla, 202 genera, and 1438 assignments on OTU level. A summary of the phylogenetic distribution of the oropharyngeal microbiome is shown in Table 1.

**TABLE 1:** Phylogenetic distribution of taxa, data based on HOMD version 2010.

Phylum	Number of classes	Number of genera	Number of OTUs	Number of taxa classified (class level)	Number of taxa unclassified (class level)
Firmicutes	5	55	519	492	27
Bacteroidetes	4	21	280	236	44
Proteobacteria	6	65	239	222	17
Actinobacteria	1	48	236	236	0
Fusobacteria	1	7	83	83	0
Spirochetes	1	2	53	53	0
TM7	1	1	25	25	0
Synergistetes	1	2	2	2	0
Verrucomicrobia	1	1	1	1	0
Total	21	202	1438	1350	88

At phylum level, 98.17% of reads were classified, with the remaining 1.82% belonging to unknown or unclassified phyla. The five main bacterial phyla present at the highest mean abundance were *Firmicutes* (mean scaled abundance per participant = 0.597, sd = 0.160), *Bacterioidetes* (mean = 0.133, sd = 0.082), *Proteobacteria* (mean = 0.118, sd = 0.147), *Actinobacteria* (mean = 0.067, sd = 0.042), and *Fusobacteria* (mean = 0.042, sd = 0.036).

### Phylum-Level Analysis

The phylum Firmicutes contained five classes and 55 genera. The class Bacilli was the largest in terms of taxa richness within the Firmicutes and contains 256 OTUs. The genus *Streptococcus* was the most frequently detected genus within the Bacilli and in the oropharynx in general, occurring at a mean relative abundance of 0.472 (sd = 0.175) across all samples. *Clostridia* and *Negativicutes* were also extremely common genera, containing 130 and 92 OTUs, respectively. Amongst the *Negativicutes*, *Veillonella* was another genus amongst the five most frequently detected genera, occurring, however, at a much lower abundance compared to *Streptococcus* (mean = 0.055, sd = 0.037). *Veillonella* and *Streptococcus* have been previously found to be highly abundant genera at many oral sites (Aas et al., 2005).

The 280 Bacteroidetes included three classes, the highly abundant Bacteroidia (154 OTUs), Flavobacteria (81), and one member of the Sphingobacteria, as well as 44 unclassified Bacteroidetes OTUs. There were 21 genera, with *Prevotella* being the largest, as well as being amongst the five most abundant genera, with a mean abundance of 0.082 (sd = 0.060).

The phylum Proteobacteria comprised five classes and 239 OTUs, including highly represented classes such as Gammaproteobacteria (122 OTUs) and Betaproteobacteria (77 OTUs), as well as the lesser represented classes such as Epsilonproteobacteria (11 OTUs), Deltaproteobacteria (seven OTUs), and Alphaproteobacteria (five OTUs), with 17 OTUs remaining unclassified. Moreover, the phylum *Proteobacteria* compromised 65 genera. The genus *Neisseria* was found in high relative abundance across all samples (mean = 0.042, sd = 0.050), being one of the five most abundant genera.

Actinobacteria comprised a total of 236 OTUs. Amongst 48 genera within the Actinobacteria, *Actinomyces* was amongst the five most abundant genera at a mean abundance of 0.039 (sd = 0.031).

The phylum Fusobacteria contained one class (Fusobacteria), comprising 83 OTUs and seven genera.

In addition to those five prevalent and abundant phyla, our data set also included, in descending abundance; the phyla Spirochetes (53 OTUs in one class and two genera), TM7 (25 OTUs in one class and one genus), Synergistetes (two OTUs in one class and two genera), Verrucomicrobia (one OTU in one class and one genus).

### Richness, Evenness, Inter and Intra Participant Variability

**FIGURE 1:**
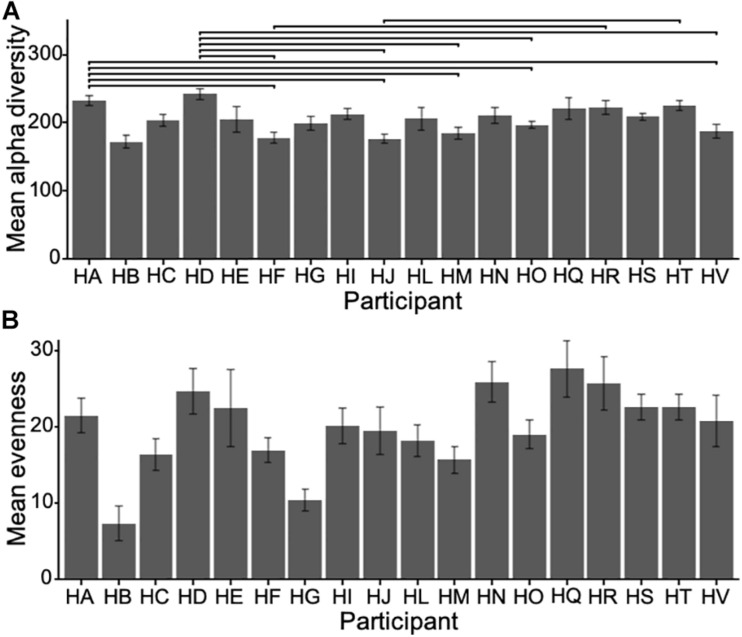
**(A)** Mean alpha diversity (i.e., count of unique OTUs in each sample) for all participants (±sd) and **(B)** mean evenness (Inverse Simpson). Horizontal bars show significant pairwise differences (*p* < 0.05) across participants. Note the difference in scale.

To compare microbial communities, we estimated alpha diversity and evenness using bootstrapped samples to a minimum of 5000 reads with replacement to subsample at a standardized sampling effort. The alpha diversity was high, with a mean of 204.55 OTUs (sd = 35.64) per participant averaged across each participant’s samples, ranging from 172.64 to 242.27 OTUs. There was a significant difference in alpha diversity across participants (one-way ANOVA *F*_17_, _295_ = 4.977, *p* < 0.001) (Figure 1A), and evenness (inverse Simpson), describing the average proportional abundances of OTUs, was also significantly different between participants (ANOVA *F*_17_, _295_ = 1.969, *p* = 0.013), with a mean of 19.83 (sd = 9.74), ranging from 7.31 to 27.62 (Figure 1B).

**FIGURE 2:**
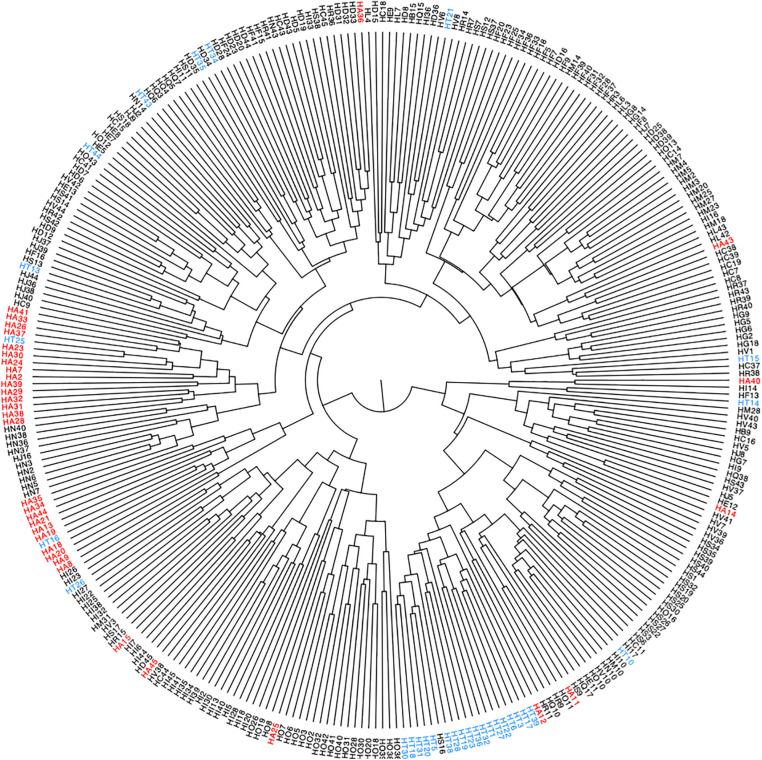
Hierarchical clustering of log10 + 1 transformed microbial abundance data of all participants across time points, with samples representing one sampling week. Highlighted are samples collected from two participants (HA in red, HT in blue), numbers indicate sampling weeks.

**FIGURE 3:**
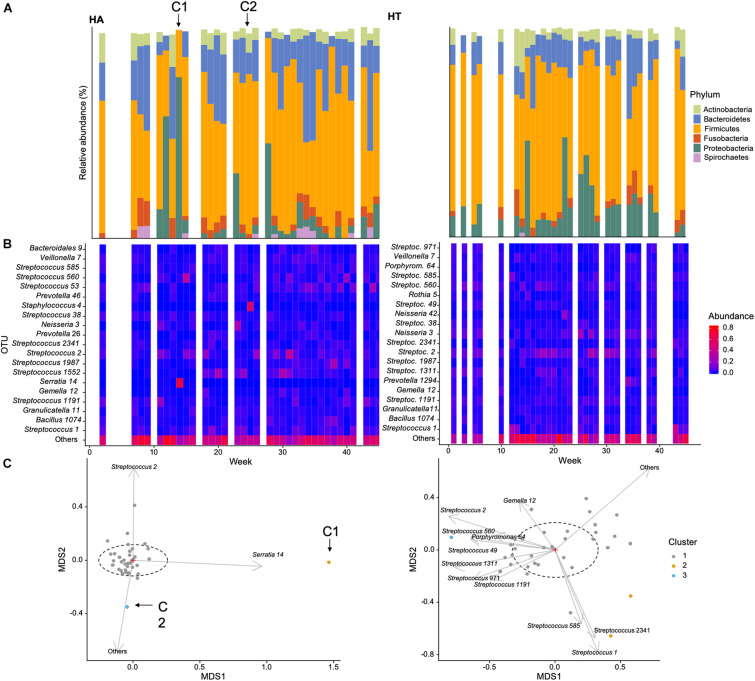
Example of temporal changes in community composition over the course of the data collection for two participants of this study: **(A)** HA (self-reporting a cold during weeks 14 annotated C1 and 26 annotated C2, C1, and C2, respectively) and **(B)** HT (self-reportedly healthy throughout the sampling period): **(A)** Relative abundance (%) of dominant phyla, **(B)** heatmap of the 20 most abundant OTUs, **(C)** non-metric multidimensional scaling ordination of bacterial community structure [dashed ellipse represents the 95% confidence interval for the centroid of each stratification group as calculated by ordiellipse (Oksanen et al., 2018)].

We used hierarchical correlation distance analysis to gain an overview of the similarity within (i.e., differences in microbial communities within a participant over the sampling interval) and between the participants (i.e., average inter-participant variability) of the study based on log10 + 1 transformed abundance data (Figure 2). Intra-participant differences were lower, ranging from a Pearson’s correlation (*r*) of 0.04 to 0.90, with a mean of 0.42 (sd = 0.116). The inter-individual similarity was, on average higher than intra-individual differences, at a range from 0.39 to 0.61, with a mean *r* of 0.52 (sd = 0.060). Most samples clustered in a participant-wise manner, indicative of a relatively consistent microbial community, but some variation of this is notable (Figure 2). For instance, samples from participant HT (highlighted in Figure 2) show marked clustering, indicative of high sample similarity during most of the sampling period. Still, some samples separated clearly from this clustering pattern (e.g., weeks 14, 15, 16, 25, and 26), which could be due to higher abundance of Proteobacteria (see below, Figure 3B).

### Multidimensional Scaling and Core Microbiome

In order to illustrate temporal changes in community abundance and composition, we selected two participants of the study, HT and HA. HT did not report any health issues throughout the study period, while HA reported cold symptoms during weeks 14 and 26 (Figure 3, C1 and C2 marked with arrows).

The relative abundance of the dominant phyla was generally more similar between time points in participant HT, and showed less variability in HA. This was specifically evident during and perhaps preceding the reported cold incident (C1) in week 14, characterized by a greater abundance of Proteobacteria (Figure 3A). This was also apparent when investigating abundance of the 20 most common OTUs (Figure 3B), with some variability in HT, but an increase in OTU 14 (genus Serratia of the Proteobacteria) during C1 for participant HA. Serratia is commonly reported to be associated with a variety of human infections (Elston and Magnuson, 1965). These findings are supported by the NMDS plot, with the sample obtained in week 14 clustering away from the main centroid, showing differences in community composition associated with increased abundance of OTU 14 (Figure 3C). The second incident of self-reported cold (C2) is also evident in the NMDS plot, correlated with a greater abundance of more rare species (i.e., Others in Figure 3C), as well as OTU 2 (Streptococcus), a common genus in the oropharynx, that can be associated with disease. This suggests that self-reported infections can be associated with changes in microbial community composition.

We also calculated mean species richness for samples with and without cold symptoms for all participants, with ANOVA (ANOVA *F*_1_, _115_ = 32.61, *p* < 0.001) indicating samples had significantly lower species richness in participants showing cold symptoms cold (mean = 149.25 OTUs, sd = 44.21) compared to those without cold symptoms (mean = 149.25, 35.75).

**FIGURE 4:**
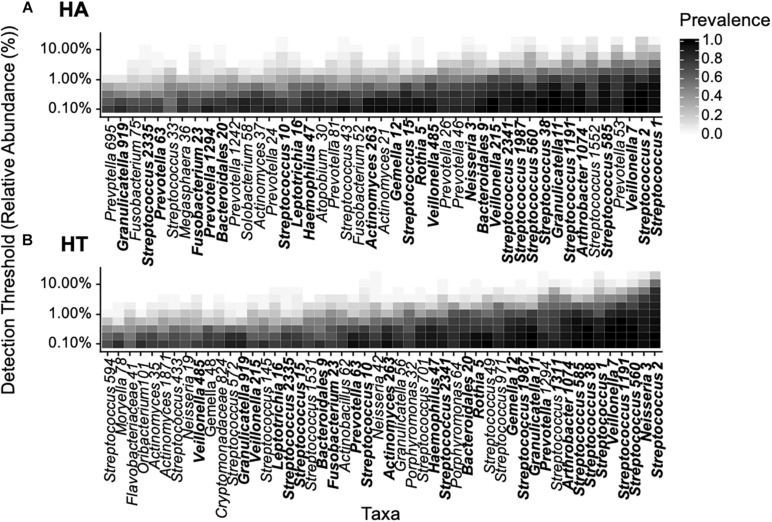
Phylogenetic core of the oropharyngeal microbiome of participants HA **(A)** and HT **(B)** based on abundance (% relative abundance) and prevalence (% occurrence in samples) in accordance with previous studies (Jalanka-Tuovinen et al., 2011), data was bootstrapped to standardize sampling effort. Relative abundance values ranged from 0.001 to 0.2 and prevalence from 5 to 100%. Overlapping OTUs between participants are highlighted bold.

We subsequently identified the core microbiome of these two individuals. Instead of initially defining specific parameter values for OTU abundance and prevalence, we followed an approach suggested previously, using adjustable parameter values, i.e., prevalence and abundance (Jalanka-Tuovinen et al., 2011). The ranges of relative abundance and prevalence values ranged from 0.001 to 0.2 and from 5 to 100%, respectively, in all samples (Figure 4). Given that the parameter values are adjustable rather than specific, the common core defined here was not a single value, but a continuum. At low levels of required relative abundance, the individual core microbiome included a relatively large number of OTUs in both participants. Similarly, if the prevalence threshold was set low, more OTUs were included in the core. We detected 49 and 45 OTUs in the cores of participant HT and HA, respectively, suggestive of a comparable core size. At detection threshold of 0.1 and prevalence of 0.5, the core microbiome consisted of 19 and 13 OTUs for HT and HA, respectively, nine of which the two participants shared, indicating relatively high similarity and overlap in the microbial core.

**FIGURE 5:**
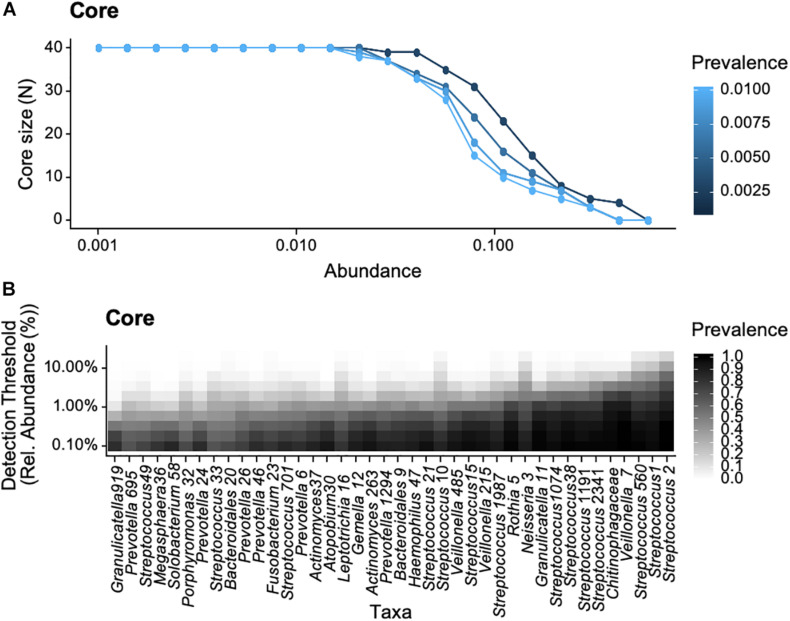
Bacterial core of the oropharyngeal microbiome from the 18 participants of the study based on selected abundance (% relative abundance) and prevalence (% occurrence in samples), data was bootstrapped to standardize sampling effort. Relative abundance values ranged from 0.001 to 0.2 and prevalence from 5 to 100%. **(A)** Core size as a function of prevalence and **(B)** bacterial taxa as a function of detection threshold and prevalence.

We subsequently explored the common core shared across all healthy individuals. The number of OTUs included in the common core decreased with increasing parameter values (i.e., increasing relative abundance and prevalence across samples, Figure 5A). Considering the range of parameters values (i.e., relative abundance values of 0.001–0.2 and prevalence of 5–100%), the common core consisted of 40 OTUs (Figure 5B). At a detection threshold of 0.1 and prevalence of 0.5, there were 12 OTUs, suggesting the common core across all participants was smaller than the individual cores discussed earlier. The common core included OTUs from the genus *Streptococci* (OTU 1 and OTU2) found in high relative abundance and prevalence across all participants (as shown earlier), as well as *Veillonella* (OTU7), occurring at lower abundances but with high prevalence across samples.

**FIGURE 6:**
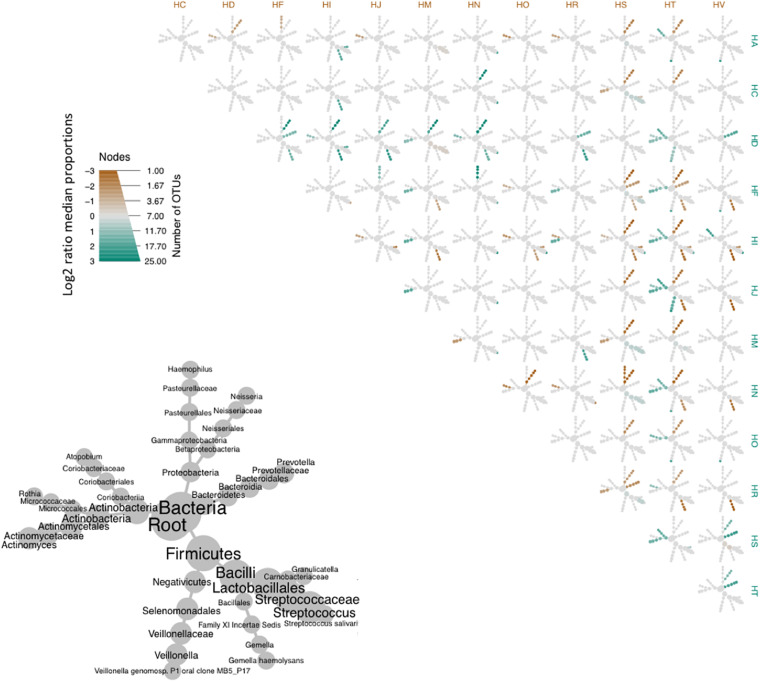
Heat tree matrix comparing OTUs of the bacterial core of the oropharyngeal microbiome from the participants of this study. Lower left-hand side diagram shows the phylogeny of the pooled data set and the sizes of the circles associated with different taxa indicate their relative abundances. Brown and cyan colors indicate significant differences across pairwise abundances, while gray represent no significant difference in relative abundance.

We then used heatmaps to explore abundance differences between OTUs in the common cores across participants (Figure 6). It was evident that core communities were generally comparable across participants. Yet, there were some participants which showed significantly different abundances of some core OTUs, such as participant HD, who showed significantly higher expression of *Prevotella*, compared to most other participants.

## Discussion

While the human oral microbiome has been extensively studied, less attention has been paid to the oropharyngeal microbiome and, to our knowledge, replicated time series data from a group of generally healthy participants has not been available until now. The oropharyngeal microbiome harbors a highly diverse bacterial community. Here, we have created a phylogenetic tree of the oropharynx comprising 1,450 OTUs, providing an in-depth picture of this specific oral site. The taxa found in high abundances, *Firmicutes* (specifically of the genus *Streptococcus*), as well as the *Bacteroidetes*, *Proteobacteria*, and *Actinobacteria*, have also been reported in other oral niches (Aas et al., 2005; Zaura et al., 2009; Dewhirst et al., 2010; Griffen et al., 2011; Segata et al., 2012; Belstrøm et al., 2016; Li and Ma, 2020). In line with previous culture-independent massive metagenomic sequencing studies (Bogaert et al., 2011; Morris et al., 2013), the genus *Streptococcus*, a very heterogeneous group comprising both commensals and pathogens, formed the dominant genus in the oral microbiome (Picard et al., 2004; Huang et al., 2011; Moon and Lee, 2016; Edouard et al., 2018), due to their ability to colonize a wide range of oral niches including epithelium, tooth surface, enamel, tonsils, or tongue (Nobbs et al., 2009). *Veillonella*, often associated with *Streptococci* (Jenkinson, 2011), is another abundant genus of the *Firmicutes*; they are a small group of generally strict anaerobic, non-fermentative Gram-negative cocci, which have been found in high abundance elsewhere in the oral microbiome, as well as in respiratory tract, small intestine, and vagina (Leuckfeld et al., 2010; Yi et al., 2014). *Prevotella* (*Bacteroidetes*) is often associated with the healthy oral microbiome but has also been linked to oral diseases (Paster et al., 2001; Huang et al., 2011).

Our longitudinal data suggested moderately high variation in community composition within participants over time, indicative of reasonable levels of stability in assemblage composition. However, the variability appears larger than that reported by others (Zoetendal et al., 1998; Scanlan et al., 2006; Jalanka-Tuovinen et al., 2011). This difference may be due to physiological or ecological characteristics of different body sites. The oral cavity is arguably a more heterogenous habitat (in terms of, for example, pH, flow rate, morphology, tissue types, or available surfaces), compared to the gastrointestinal tract resulting in greater availability of distinct habitats (Zaura et al., 2009; Jenkinson, 2011; Simon-Soro et al., 2013). Further, the higher rate of immigration/emigration of taxa within the oral cavity (Dewhirst et al., 2010) may require a wider range of adaptations, resulting in the evolution a greater number of functionally interchangeable taxa. As expected, inter-individual variation was higher than intra-individual variation, suggesting that individuals were characterized by having varying, but characteristic taxa. This finding is in line with conclusions drawn by a number of other studies (Costello et al., 2009; Caporaso et al., 2011; Stahringer et al., 2012; Flores et al., 2014; Cameron et al., 2015; Utter et al., 2016), from oral data (saliva, tongue, and dental plaque), where inter-individual variation in microbial assemblage was greater than intra-individual variation. This may be due to extrinsic factors including diet, medication, or intrinsic factors such as immune system differences.

The microbiome of the oropharynx showed high alpha diversity, the values here were similar to those reported for other oral sites (Zaura et al., 2009; Huang et al., 2011; Diaz et al., 2012; Thomas et al., 2014; Zheng et al., 2015; Edouard et al., 2018), indicative that our data set captures the diversity of the oropharynx. There was considerable variation across individuals, perhaps due to factors discussed previously, such as lifestyle (e.g., diet, oral hygiene, and travel), inherent genetic variation, immune system or random colonization events. The results indicated the dominance of few bacterial OTUs (such as *Streptococcus*) (Moon and Lee, 2016).

By investigating the community composition of two participants in more detail, it became apparent that microbial community composition on the phylum level was generally similar over time, suggestive of stability, and showed relatively high commonality across the two participants, as discussed elsewhere (Moon and Lee, 2016). Yet, our results suggest that the microbial communities in the two participants are personalized, varying in a participant-specific (Flores et al., 2014; Edouard et al., 2018) manner (Flores et al., 2014; Edouard et al., 2018). As expected, there was much variation across those two participants at OTU level (e.g., Figures 4A,B), which similarly to the results of the Pearson’s correlation findings, showed high inter-participant variability in microbial community assemblage structure. It is worth noting that there were indications of changes in community composition in participant HA, when a cold was reported, particularly evident in the NMDS plot, and correlated to changes in abundance of specific OTUs (as seen by overlaid vectors). This suggests we are able to detect and describe specific changes in abundance and assemblage composition in disease states. This may also allow us to determine which OTUs are disease-associated, using them as biomarkers for future studies. When comparing across all participants, we also found a significant decrease in species richness in participants reporting cold symptoms, as evidenced by others (Yi et al., 2014; Korten et al., 2016; Piters et al., 2016; Edouard et al., 2018). These reductions are often associated with increasing abundances of pathogenic species (e.g., *Staphylococcus aureus*, *Streptococcus pneumoniae*, *Moraxella catarrhalis*, or *Haemophilus influenzae*). To gain a deeper understanding in the species-specific changes, species (rather than genus-level) resolution is required.

A key question of the Human Microbiome Project is, whether it is possible to identify a core microbiome, consisting of common organisms inhabiting similar habitats or body sites that exist in the majority or all humans (Hamady and Knight, 2009; Shade and Handelsman, 2012; de Cárcer, 2018). The identification of this core microbiome has followed a number of approaches. One such approach is to identify shared OTUs (or phylotypes) across samples, reporting their overlap from presence/absence data (Turnbaugh et al., 2007). Thus, if found in a vast majority of participants, an OTU is defined as a core taxon, irrespective of it occurring in high or low abundances. An alternative and perhaps more stringent definition of the core microbiome is where OTUs have to occur in all participants (i.e., in 100% of all samples) (Huse et al., 2012). Previously, the oropharynx core microbiome has been identified on three participants by Zaura et al. (2009), and on a greater sample size by Huse et al. (2012), defining core as taxa shared in 95% of individuals. Neither of these studies, however, have included time-series information. Time series data has the potential to provide some general insights before specifically defining microbiome as that consistently observed across time points and shared across individuals (Shade and Handelsman, 2012). Our approach here addressed the individual core microbiome within two participants and the common core across participants in a conceptual definition across values of relative abundance and prevalence, and then in a concrete manner by selecting specific thresholds. The common core included fewer OTUs than the individual core, which is perhaps not surprising given that it includes a greater number of samples. The common core may consist of taxa selected during the co-evolution of host and microbiome, and once obtained, it may be possible to catalog the functional roles of those microbes. Our results support that many of the bacterial taxa are present in the oropharynx across individuals, often in similar abundances as indicated by the heat maps. Of those, Firmicutes (specifically *Streptococcus* sp.) are highly represented in the two individual and common cores, similar to other studies (Zaura et al., 2009; Eren et al., 2014; de Cárcer, 2018). In identifying a core, we are moving further toward resolving some of the immense complexity and the significant intra and individual variability in the microbial communities, which currently hamper our ability to resolve differences between group and input models without overparameterizing.

This work is the first to comprehensively define the phylogeny of the human oropharynx based on 18 participants sampled over a period of 40 weeks. We extend our understanding of the species richness and evenness of the healthy oropharyngeal microbiome, and its common core, allowing us to move closer toward being able to define the healthy oropharyngeal microbiome.

## Data Availability Statement

The following data: otu_table.biom, otus.fasta, otus.tre and meta_data.csv file are available on Github (https://github.com/umerijaz/pharynxmicrobiome).

## Ethics Statement

The studies involving human participants were reviewed and approved by University of Glasgow Ethics Committee. The patients/participants provided their written informed consent to participate in this study.

## Author Contributions

AR collected the data and conducted the sequencing. LB conducted the analysis and wrote the manuscript with supervision from JL and support from UI and TE. LB took the lead in writing the manuscript. All authors discussed the results and contributed to the final manuscript.

## Conflict of Interest

The authors declare that the research was conducted in the absence of any commercial or financial relationships that could be construed as a potential conflict of interest.

## Footnotes

1 http://drive5.com/uchime/uchime_download.html

2 http://microbiomeutil.sourceforge.net/
