# Supplementary material for: A Longitudinal Study of the Human Oropharynx Microbiota Over Time Reveals a Common Core and Significant Variations With Self-Reported Disease
Source: Front Microbiol. 2021 Jan 21;11:573969. doi: 10.3389/fmicb.2020.573969 (PMC7861042; doi:10.3389/fmicb.2020.573969)
Supplement: Supplementary file 1 [file Data_Sheet_1.docx]

Supplementary Material

# Supplementary Materials: QIA-AMP DNA extraction protocol

1. Store swabs in transport medium during transport to the laboratory.
If swabs are not processed immediately they should be stored at 2-8°C for up to 24 hours. Any period longer than this will be require storage at -20°C.

2. All swabs should be vortexed to ensure dispersal of microorganisms from swab to fluid.

3. Remove swab and place in a 2ml microcentrifuge tube. Centrifuge swab tip at 5,000 x g or 8,000rpm to remove any remaining fluid in the tube. After centrifugation remove swab tip and transfer 1ml of suspension fluid into the same 2ml microcentrifuge tube and centrifuge for 10 minutes at full speed (20,000 x g; 14,000rpm).

4. Suspend pellet in 180μl of enzymatic lysis buffer (20mg/ml lysozyme or 200μg/ml lysostaphin; 20mM TrisHCl, pH 8.0; 2mM EDTA; 1.2% Triton). If there is no pellet formed or pellet formation is very small, remove as much supernatant as possible without touching the bottom of the tube and add 180μl of enzymatic lysis buffer to the same microcentrifuge tube.

5. Incubate for at least 30 minutes at 37°C.

6. Add 20μl Proteinase K and 200μl Buffer AL. Mix by vortexing for 10 seconds.

7. Incubate at 56°C for 1 hour. If using a heat block, vortex the tube for 10 seconds every 10 minutes.

8. Centrifuge for a few seconds to remove drops from inside the lid.

9. Incubate the 2ml microcentrifuge at 70°C for 10 minutes. If using a heat block vortex the tube for 10 seconds every 3 minutes to improve lysis.

10. Centrifuge for a few seconds to remove drops from inside the lid.

11. Add 200μl ethanol (96-100%) to the sample and mix by vortexing for 10 seconds.

12. Centrifuge for a few seconds to remove drops from inside the lid.

13. Carefully transfer the lysate from the 2ml microcentrifuge tube into a QIAamp Mini spin column (2ml collection tube). Close the cap and centrifuge at 8000 x g (6000rpm) for 1 minute (if the lysate has not completely passed through the 2ml column after centrifugation, centrifuge at a higher speed until the QIAamp Mini spin column is empty). Place the QIAamp Mini spin column in a clean 2ml collection tube and discard the tube containing the filtrate. Transfer any remaining lysate from the 2ml microcentrifuge tube and repeat as above.

14. Add 500μl of Buffer AW1 to the QIAamp Mini spin column. Close the cap and centrifuge at 8000 x g (6000rpm) for 1 minute. Place the QIAamp Mini spin column in a clean 2ml collection tube and discard the tube containing the filtrate.

15. Add 500μl of Buffer AW2 to the QIAamp Mini spin column. Close the cap and centrifuge at 8000 x g (6000rpm) for 1 minute. Place the QIAamp Mini spin column in a clean 2ml collection tube and discard the tube containing the filtrate.

16. Centrifuge at full speed (20,000 x g; 14,000rpm) for 3 minutes to dry the membrane.

17. Place the QIAamp Mini spin column in a clean 1.5ml microcentrifuge tube and discard the collection tube containing the flow-through. Carefully open the lid of the QIAamp Mini spin column and apply 50μl Buffer AE (having 2 centrifugation steps of 25μl Buffer AE and a final re-elution step increases DNA yield).

18. Close the lid and incubate at room temperature for 5 minutes. Centrifuge at full speed (20,000 x g or 14,000rpm) for 1 minute.

19. Qubit.

20. Run 10μl of DNA extract with 2μl loading buffer on a gel to check purity (best to run extractions on a 1% gel -1g agarose to 100ml TAE or TBE.)
If using Bioline mix reagents loading gel does not need to be used on the gel.

21. Run at 100v for 50 minutes.

22. Store DNA at -20°C until required.

# Supplementary Materials: Production of an rDNA clone library

**Production of PCR products:**

1. Set up the following 50μl PCR reaction:

25μl Bioline PCR mix
1μl primer (forward and reverse) at 12.5pmol each 2μl DNA template
21μl water
Total Volume 50μl

**The PCR reaction should run under the following conditions:**

Initial denaturation - 95°C for 5 minutes
35 cycles of denaturation - 94°C for 1 minute Annealing - 62°C for 1 minute
Extension - 72°C for 1 minute
Final Extension - 72°C for 10 minutes

Check the PCR product by agarose gel electrophoresis to ensure production of a single discrete band.

**QIA gel extraction kit protocol – extraction and purification of DNA from agarose gels:**

1. Cut DNA fragment from the agarose gel with a scalpel.

2. Weigh the gel slice in a colourless tube.

Add 3 volumes of buffer QG to 1 volume of gel (100mg - 100μl).
For >2% agarose gels, add 6 volumes of Buffer QG. The maximum amount of gel slice per QIAquick column is 400mg; for gel slices >400mg use more than one QIAquick column.

3. Incubate at 50°C for 10 minutes.
Vortex every 2-3 minutes to dissolve the gel.

4. After the gel slice has dissolved completely, check that the colour of the mixture is yellow (similar to Buffer QG without dissolved agarose). If the colour of the mixture is orange or violet, add 10μl of 3M sodium acetate, pH 5.0, and mix. The colour of the mixture will turn yellow.

5. Add 1 gel volume of isopropanol to the sample and mix (if the agarose gel slice is 100mg, add 100μl isopropanol).

6. Place a QIAquick spin column in a provided 2ml collection tube.

7. To bind DNA, apply the sample to the QIAquick column and centrifuge (1 minute). Discard flow-through and place QIAquick column back in the same collection tube.

8. Add 300ml of buffer QG to QIAquick column and centrifuge for 1 minute to remove all traces of agarose.

9. To wash, add 300ml of Buffer PE to QIAquick column (stand for 2-5 minutes) and centrifuge for 1 minute.

10. Discard the flow-through and centrifuge the QIAquick column for an additional 1 minute at > 10,000 x g (13,000rpm).

11. Place QIAquick column into a clean 1.5ml microcentrifuge tube.

12. To elute DNA, add 30μl of Buffer EB (10mM TrisCl, pH 8.5) to the centre of the QIAquick membrane and centrifuge the column for 1 minute at maximum speed. For an increased DNA concentration, let the column stand for 5 minutes, and then centrifuge for 1 minute.

**Production of lunia bertani broth and agar plates:**

For 500ml broth:

5g – Tryptone
2.5g – Yeast agar
5g – NaCl
Add water to make a total volume of 500ml and autoclave. When broth has cooled add 5ml of Kanamycin.

For 200ml agar plates:

Add 3g of agar in 200ml of water and autoclave. When agar has cooled add 2ml of Kanamycin. Flame the bottle top using a Bunsen burner. Pour into plates.

Flame agar plates to get rid of any bubbles. Let plates harden and store in fridge.

**Performing TOPO cloning reaction:**

1. Set up the following reaction: Fresh PCR product – 0.5 - 4μl
Salt solution - 1μl
Water – add to a total volume of 5μl TOPO vector - 1μl

Total volume - 6μl
The cloning reaction can be stored overnight at -20°C.

2. Mix the reaction gently and incubate for 30 minutes at room temperature (22°C - 23°C).

3. Place the reaction on ice ready for the next step.

**Transforming cells:**
1. Warm selective plates to 37°C prior to spreading.

2. Add 2μl of the TOPO cloning reaction into a vial of OneShot®Chemically competent E. coli and mix gently.

3. Incubate on ice for 30 minutes.

4. Heat shock the cells for 30 seconds at 42°C without shaking.

5. Immediately transfer the tubes to ice.

6. Add 250μl of S.O.C medium.

7. Cap the tube tightly and shake the tube horizontally (200rpm) at 37°C for 1 hour.

8. Spread 10-50μl from each transformation on a pre-warmed selective plate. To ensure even spreading of small volumes, add 20μl of S.O.C medium (plate two different volumes to ensure that at least one plate will have well-spaced colonies). Spread at least 5 plates in total.

9. Incubate plates at 37°C (ampicillin plate should produce colonies within 8 hours whereas kanomycin plates should be incubated overnight).

10. An efficient TOPO cleaning reaction should produce several hundred colonies. Pick 100-200 colonies for analysis.

**Analysing transformants:**
1. Pick 100-150 colonies for analysis.

2. Aliquot 500μl of LB kanamycin into a 96 deep well plate.

3. By the flame of a Bunsen burner, using a pipette and clean tip, pick up a single colony and inoculate a well in the deep well plate. Incubate overnight with shaking at 37°C at 200rpm.

4. Screen the colonies the next day for the correct insert.

5. Remove 200μl of each sample in the deep well plate (96 samples altogether) to be amplified using the M13F and M13R primers.

6. Set up the following PCR reaction to ensure the samples take up the vector: Biomix – 10μl
M13F primer – 0.5μl
M13R primer – 0.5μl

Water - 7μl
DNA - 2μl
Total volume = 20μl

The reaction should run for 25-30 cycles at the following conditions: 95°C – 5 minutes
94°C – 1 minute
55°C – 1 minute

72°C – 1 minute 72°C – 10 minutes

Run a gel at 100v for 60 minutes to ensure that each sample has taken up the plasmid.

**Restriction digest:**
1. Do a restriction digestion on all 96 samples.

2. Set up the following reaction: PCR reaction - 5μl
Hae 111 restriction digest – 0.1μl Water – 8.4μl

Buffer – 1.5μl
Total volume = 15μl

3. Centrifuge tubes and incubate at 37°C for 4 hours.

4. Prepare a 2% gel for all 96 isolates (add 3μl of loading dye to the 15μl reaction). Run the gel at 100v for 90 minutes.

5. From the gel group isolates into OTUs depending on the same banding patterns. Each type of OTU will be sent for Sanger Sequencing.

6. Grow each OTU selected for Sanger Sequencing overnight in 5ml of LB and kanamycin (37°C, 200rpm for 20-24 hours).

**Plasmid extractions:**
1. Extract the DNA from the plasmid using the Invitrogen Purelink Quick Plasmid MiniPrep Kit.

2. Centrifuge 1–5ml of the overnight LB-culture (5 minutes at 5000rpm).

3. Add 250μl Resuspension Buffer (R3) with RNase A to the cell pellet and resuspend the pellet until it is homogeneous.

4. Add 250μl Lysis Buffer (L7). Mix gently by inverting the capped tube until the mixture is homogeneous. Do not vortex. Incubate the tube at room temperature for 5 minutes.

5. Add 350μl Precipitation Buffer (N4). Mix immediately by inverting the tube or for large pellets by vigorously shaking the tube, until the mixture is homogeneous. Do not vortex. Centrifuge the lysate at >12,000 × g for 10 minutes.

6. Load the supernatant from step 4 onto a spin column in a 2ml wash tube. Centrifuge the column at 12,000 × g for 1 minute. Discard the flow-through and place the column back into the wash tube.

7. Add 500μl Wash Buffer (W10) with ethanol to the column. Incubate the column for 1 minute at room temperature. Centrifuge the column at 12,000 × g for 1 minute. Discard the flow-through and place column back into the wash tube.

8. Add 700μl Wash Buffer (W9) with ethanol to the column. Centrifuge the column at 12,000 × g for 1 minute. Discard the flow-through and place the column into the wash tube. Centrifuge the column at 12,000 × g for 1 minute. Discard the wash tube with the flow-through.

9. Place the spin column in a clean 1.5ml recovery tube. Add 75μl of preheated TE Buffer (TE) to the centre of the column. Incubate the column for 1 minute at room temperature.

10. Centrifuge the column at 12,000 × g for 2 minutes. The recovery tube contains the purified plasmid DNA. Discard the column. Store plasmid DNA at 4°C (short-term) or store the DNA in aliquots at −20°C (long-term).

**Long term storage:**

1. Streak out the original colony on LB plates.

2. Isolate a single colony and inoculate into 1-2ml of LB.

3. Grow overnight until culture is saturated.

4. Mix 0.85ml of culture with 0.15ml of sterile glycerol and transfer to a cryovial.

5. Store at -80°C.

# Supplementary Materials: QIAGEN QIA gel extraction kit protocol

1. Cut the DNA fragment from the agarose gel with a scalpel.

2. Weigh the gel slice in a colourless tube. Add 3 volumes of buffer QG to 1 volume of gel (100ml - 100μl). For example, add 300μl of Buffer QG to each 100 mg of gel. For >2% agarose gels, add 6 volumes of Buffer QG. The maximum amount of gel slice per QIAquick column is 400mg; for gel slices >400mg use more than one QIAquick column.

3. Incubate at 50°C for 10 minutes.

4. After the gel slice has dissolved completely, check that the colour of the mixture is yellow (similar to Buffer QG without dissolved agarose). If the colour of the mixture is orange or violet, add 10μl of 3M sodium acetate, pH 5.0, and mix. The colour of the mixture will turn to yellow.

5. Add 1 gel volume of isopropanol to the sample and mix (if the agarose gel slice is 100mg, add 100μl isopropanol).

6. Place a QIAquick spin column in a provided 2ml collection tube.

7. To bind DNA, apply the sample to the QIAquick column and centrifuge for 1

minute.

8. Discard flow-through and place QIAquick column back in the same collection tube.

9. Add 0.5ml of Buffer QG to QIAquick column and centrifuge for 1 minute to remove all traces of agarose.

10. To wash, add 0.75ml of Buffer PE to the QIAquick column and centrifuge for an additional 1 minute.

11. Discard the flow-through and centrifuge the QIAquick column for an additional 1 minute at > 10,000 x g (13,000rpm).

12. Place QIAquick column into a clean 1.5ml microcentrifuge tube.

13. To elute DNA, add 50μl of Buffer EB (10mM TrisCl, pH 8.5) to the centre of the QIAquick membrane and centrifuge the column for 1 minute at maximum speed. For an increased DNA concentration, add 30μl elution buffer to the centre of the QIAquick membrane, let the column stand for 1 minute, and then centrifuge for 1 minute.

**
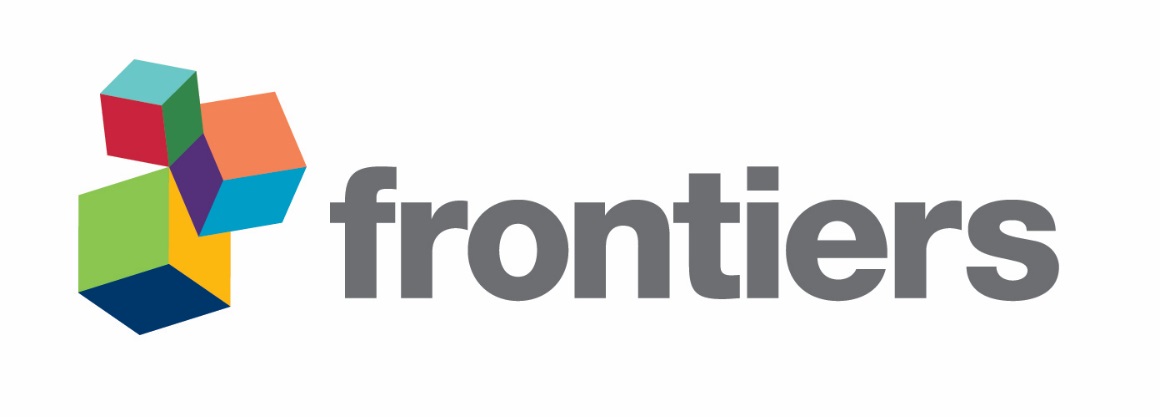
**
